# The RNA processing enzyme polynucleotide phosphorylase negatively controls biofilm formation by repressing poly-*N*-acetylglucosamine (PNAG) production in *Escherichia coli* C

**DOI:** 10.1186/1471-2180-12-270

**Published:** 2012-11-21

**Authors:** Thomas Carzaniga, Davide Antoniani, Gianni Dehò, Federica Briani, Paolo Landini

**Affiliations:** 1 Department of Biosciences, University of Milan, Via Celoria 26, Milan, 20133, Italy

**Keywords:** Biofilm, RNA processing, Degradosome, EPS, Cell adhesion, PNPase

## Abstract

**Background:**

Transition from planktonic cells to biofilm is mediated by production of adhesion factors, such as extracellular polysaccharides (EPS), and modulated by complex regulatory networks that, in addition to controlling production of adhesion factors, redirect bacterial cell metabolism to the biofilm mode.

**Results:**

Deletion of the *pnp* gene, encoding polynucleotide phosphorylase, an RNA processing enzyme and a component of the RNA degradosome, results in increased biofilm formation in *Escherichia coli*. This effect is particularly pronounced in the *E*. *coli* strain C-1a, in which deletion of the *pnp* gene leads to strong cell aggregation in liquid medium. Cell aggregation is dependent on the EPS poly-*N*-acetylglucosamine (PNAG), thus suggesting negative regulation of the PNAG biosynthetic operon *pgaABCD* by PNPase. Indeed, *pgaABCD* transcript levels are higher in the *pnp* mutant. Negative control of *pgaABCD* expression by PNPase takes place at mRNA stability level and involves the 5’-untranslated region of the *pgaABCD* transcript, which serves as a *cis*-element regulating *pgaABCD* transcript stability and translatability.

**Conclusions:**

Our results demonstrate that PNPase is necessary to maintain bacterial cells in the planktonic mode through down-regulation of *pgaABCD* expression and PNAG production.

## Background

Most bacteria can switch between two different lifestyles: single cells (planktonic mode) and biofilms, *i*.*e*., sessile microbial communities. Planktonic and biofilm cells differ significantly in their physiology and morphology and in their global gene expression pattern [1-3]. Extensive production of extracellular polysaccharides (EPS) represents a defining feature of bacterial biofilms; EPS are the major constituent of the so-called “biofilm matrix”, which also includes cell surface-associated proteins and nucleic acids [4,5]. In addition to constituting the material embedding biofilm cells and to being a main determinant for surface attachment, the EPS are responsible for cell resistance to environmental stresses such as desiccation [6] and to predation by bacteriophages [7]. In several bacterial species, EPS are also required for swarming motility [8,9].

Expression of genes involved in EPS biosynthesis is controlled by complex regulatory networks responding to a variety of environmental and physiological cues, including stress signals, nutrient availability, temperature, etc. [10-13]. Regulation of EPS production can take place at any level, *i*.*e*., transcription initiation, mRNA stability, and protein activity. For instance, the *vps* genes, involved in EPS biosynthesis in *Vibrio cholerae*, are regulated at the transcription level by the CytR protein, in response to intracellular pyrimidine concentrations [14]. The RsmA protein negatively regulates EPS production in *Pseudomonas aeruginosa* by repressing translation of the *psl* transcript [15]. Finally, cellulose production in *Gluconacetobacter xylinum* and in various enterobacteria requires enzymatic activation of the cellulose biosynthetic machinery by the signal molecule cyclic-di-GMP (c-di-GMP) [16,17], a signal molecule which plays a pivotal role as a molecular switch to biofilm formation in Gram negative bacteria [18]. The great variety of regulatory mechanisms presiding to EPS biosynthesis, and the role of c-di-GMP as signal molecule mainly devoted to its control, underline the critical importance of timely EPS production for bacterial cells.

Polynucleotide phosphorylase (PNPase) plays an important role in RNA processing and turnover, being implicated in RNA degradation and in polymerization of heteropolymeric tails at the 3’-end of mRNA [19,20]. PNPase is an homotrimeric enzyme that, together with the endonuclease RNase E, the DEAD-box RNA helicase RhlB, and enolase, constitute the RNA degradosome, a multiprotein machine devoted to RNA degradation [21,22]. Despite the crucial role played by PNPase in RNA processing, the *pnp* gene is not essential; however, *pnp* inactivation has pleiotropic effects, which include reduced proficiency in homologous recombination and repair [23,24], inability to grow at low temperatures [25] and inhibition of lysogenization by bacteriophage P4 [26]. Moreover, lack of PNPase affects stability of several small RNAs, thus impacting their ability to regulate their targets [27].

In this work, we show that deletion of the *pnp* gene results in strong cell aggregation and biofilm formation, due to overproduction of the EPS poly-*N*-acetylglucosamine. Increased biofilm formation was observed both in *E*. *coli* MG1655 and C-1a strains, being more pronounced in the latter. We demonstrate that PNPase negatively controls expression of the PNAG biosynthetic operon *pgaABCD* at post-transcriptional level, thus acting as a negative determinant for biofilm formation. Our observation that PNPase acts as an inhibitor of biofilm formation is consistent with previous findings highlighting the importance of regulation of EPS production and biofilm formation at mRNA stability level [28].

## Methods

### Bacteria and growth media

Bacterial strains and plasmids are listed in Table 1. *E*. *coli* C-1a is a standard laboratory strain [29], whose known differences with *E*. *coli* MG1655 reside in its restriction/modification systems [30] and in the presence of a functional *rph* gene, encoding ribonuclease PH, which, in contrast, is inactivated by a frameshift mutation in *E*. *coli* MG1655 [31]. For strain construction by λ Red-mediated recombination [32], if not otherwise indicated, the parental strains were transformed with DNA fragments obtained by PCR using either pKD3 (for amplification of DNA fragments carrying chloramphenicol-resistance cassettes) or pKD13 (for DNA fragments carrying kanamycin-resistance cassettes) as template. The sequences of oligonucleotides utilized in this work are reported in Additional file 1: Table S1. Bacterial cultures were grown in the following media: LD (10 g/l tryptone, 5 g/l yeast extract, 5 g/l NaCl); M9 (82 mM Na_2_HPO_4_, 24 mM KH_2_PO_4_, 85 mM NaCl, 19 mM NH_4_Cl, 1 mM MgSO_4_, 0.1 mM CaCl_2_, 0.1 μg/ml thiamine); M9/sup (M9 supplemented with 0.25 g/l tryptone, 0.125 g/l yeast extract, 0.125 g/l NaCl). Unless otherwise stated, 0.4% glucose was added to give either M9Glu or M9Glu/sup media. When needed, media were supplemented with 100 μg/ml ampicillin.

**Table 1:** Bacterial strains and plasmids

**Strains**	**Relevant Genotype**	**Origin or reference**
C-1a	*E*. *coli C*, prototrophic	[40]
C-5691	Δ*pnp*-*751*	[41]
C-5928	Δ*bcsA*::*cat*	by P1 HTF AM72 transduction into C-1a
C-5929	Δ*pnp*-*751* Δ*bcsA*::*cat*	by P1 HTF AM72 transduction into C-5691
C-5930	Δ*csgA*::*cat*	by P1 HTF AM70 transduction into C-1a
C-5931	Δ*pnp*-*751* Δ*csgA*::*cat*	by P1 HTF AM70 transduction into C-5691
C-5932	Δ*pgaA*::*cat*	by P1 HTF AM56 transduction into C-1a
C-5933	Δ*pnp*-*751* Δ*pgaA*::*cat*	by P1 HTF AM56 transduction into C-5691
C-5934	Δ*wcaD*::*tet*	by P1 HTF AM105 transduction into C-1a
C-5935	Δ*pnp*-*751* Δ*wcaD*::*tet*	by P1 HTF AM105 transduction into C-5691
C-5936	Δ*pgaC*::*kan*	by P1 HTF JW1007 transduction into C-1a
C-5937	Δ*pnp*-*751* Δ*pgaC*::*kan*	by P1 HTF JW1007 transduction into C-5691
C-5938	Δ*csrA*::*kan*	From C-1a by λ Red-mediated recombination; primers: FG2624 and FG2625
C-5940	Δ*csrB*::*kan*	From C-1a by λ Red-mediated recombination; primers: FG2524 and FG2525
C-5942	Δ*pnp*-*751* Δ*csrB*::*kan*	From C-5691 by λ Red-mediated recombination; primers: FG2524 and FG2525.
C-5944	Δ*csrC*::*cat*	From C-1a by λ Red-mediated recombination; primers: FG2585 and FG2586.
C-5946	Δ*pnp*-*751* Δ*csrC*::*cat*	From C-5691 by λ Red-mediated recombination; primers: FG2585 and FG2586.
C-5948	Δ*csrB*::*kan* Δ*csrC*::*cat*	by P1 HTF C-5940 transduction into C-5944
C-5950	Δ*pnp*-*751* Δ*csrB*::*kan* Δ*csrC*::*cat*	by P1 HTF C-5940 transduction into C-5946
C-5952	Δ*csrD*::*cat*	From C-1a by λ Red-mediated recombination; primers: PL674 and PL675.
C-5954	Δ*pnp*-*751* Δ*csrD*::*cat*	From C-5691 by λ Red-mediated recombination; primers: PL674 and PL675.
C-5960	Δ*mcaS*::*kan*	From C-1a by λ Red-mediated recombination; primers: FG2755 and FG2756.
C-5962	Δ*pnp*-*751* Δ*mcaS*::*kan*	From C-5691 by λ Red-mediated recombination; primers: FG2755 and FG2756.
JW1007	BW25113 Δ*pgaC*::*kan*	[68]
AM56	MG1655 Δ*pgaA*::*cat*	[69]
AM70	MG1655 Δ*csgA*::*cat*	[69]
AM72	MG1655 Δ*bcsA*::*cat*	[69]
AM105	MG1655 Δ*wcaD*::*tet*	From MG1655 by λ Red-mediated recombination with a DNA fragment obtained by PCR of tet10 cassette of EB 1.3 with primers PL372 and PL373.
EB 1.3	MG1655 *rpoS*::Tn10-tet	[33]
**Plasmids and phage**	**Relevant characteristics**	**Reference**
pBAD24	Amp^R^, ColE1	[70]
pBAD24-Δ1	pBAD24 derivative with a modified polylinker; carries an unique *Nco*I site overlapping the *araBp* transcription start	this work
pBADpnp	pBAD24 derivative; harbours an EcoRI-HindIII fragment of pEJ01 that carries the *pnp* gene	this work
pBADrnb	pBAD24 derivative; harbours an HindIII-XbaI fragment of pFCT6.9 that carries the *rnb* gene	this work
pBADrnr	pBAD24-Δ1 derivative; harbours the *rnr* gene (obtained by PCR on MG1655 DNA with FG2474-FG2475 oligonucleotides) between NcoI-HindIII sites	this work
pΔLpga	pJAMA8 derivative, harbours the -116 to +32 region relative to the *pgaABCD* transcription start site cloned into the SphI/XbaI sites	this work
pEJ01	carries a His-tagged *pnp* allele	[71]
pFCT6.9	carries a His-tagged *rnb* allele	[72]; received from Cecilia Arraiano
pGZ119HE	*ori*V_ColD_; Cam^R^	[73]
pJAMA8	Amp^R^, ColE1; *luxAB* based promoter-probe vector.	[37]
pLpga1	pJAMA8 derivative, harbours the -116 to +234 region relative to the *pgaABCD* transcription start site cloned into the SphI/XbaI sites.	this work
pLpga2	pJAMA8 derivative, harbours a translational fusion of *pgaA* promoter, regulatory region and first 5 codons of *pgaA* (-116 to +249 relative to transcription start site) with *luxA* ORF (Open Reading Frame).	this work
pTLUX	pJAMA8 derivative, harbours *ptac* promoter of pGZ119HE cloned into the SphI/XbaI sites.	this work
P1 HTF	High transduction frequency phage P1 derivative	[74]; received from Richard Calendar

### Cell aggregation and adhesion assays

Cell aggregation was assessed as follows: overnight cultures grown in LD at 37°C on a rotatory device were diluted 50-fold in 50 ml of M9Glu/sup in a 250 ml flask. The cultures were then incubated at 37°C with shaking at 100 rpm. Cell adhesion to the flask walls was assessed in overnight cultures grown in M9Glu/sup medium at 37°C. Liquid cultures were removed and cell aggregates attached to the flask glass walls were stained with crystal violet for 5 minutes to allow for better visualization. Quantitative determination of surface attachment to polystyrene microtiter wells was carried out using crystal violet staining as previously described [33]. Binding to Congo red (CR) was assessed in CR agar medium (1% casamino acid, 0.15% yeast extract, 0.005% MgSO_4_, 2% agar; after autoclaving, 0.004% Congo red and 0.002% Coomassie blue). Overnight cultures in microtiter wells were replica plated on CR agar plates, grown for 24 h at 30°C, and further incubated 24 h at 4°C for better detection of staining.

### Gene expression determination

RNA extraction, Northern blot analysis and synthesis of radiolabelled riboprobes by *in vitro* transcription with T7 RNA polymerase were previously described [34,35]. The DNA template for PGA riboprobe synthesis was amplified by PCR on C-1a genomic DNA with oligonucleotides FG2491/39 and FG2492/22. Autoradiographic images of Northern blots were obtained by phosphorimaging using ImageQuant software (Molecular Dynamics). Quantitative (real time) reverse transcriptase PCR (quantitative RT-PCR) was performed as described [33]. Oligonucleotides PL101/21 and PL102/19 were used for 16S rRNA reverse transcription and PCR amplification. mRNA half-lives were estimated as described [36] by regression analysis of mRNA remaining (estimated by real time PCR) versus time after rifampicin addition. Luciferase assays were performed as in [37]. Oligonucleotides utilized for Northern blot, real time PCR, and construction of reporter plasmids are listed in Additional file 1: Table S1.

### PNAG detection

PNAG production was determined as described [38]. Bacteria were grown overnight in 3 ml of M9 Glu/sup medium at 37°C. Cells were collected by centrifugation and diluted in Tris-buffered saline [20 mM Tris–HCl, 150 mM NaCl (pH 7.4)] to an OD_600_ = 1.5. 1ml of suspension was centrifuged at 10,500 x *g*, resuspended in 300 μl of 0.5 M EDTA (pH 8.0), and incubated for 5 min at 100°C. Cells were removed by centrifugation at 10,500 x *g* for 6 min and 100 μl of the supernatant was incubated with 200 μg of proteinase K for 60 min at 60°C. Proteinase K was heat-inactivated at 80°C for 30 min. The solution was diluted 1:3 in Tris-buffered saline and 10 μl was spotted onto a nitrocellulose filter using a Dot-blot apparatus (Bio-Rad). The filter was saturated for about 2 hours in 0.1 M Tris–HCl (pH 7.5), 0.3 M NaCl, 0.1% Triton (Sigma Aldrich) and 5% milk and then incubated overnight at 4°C with a 1:1,000 dilution of purified PNAG antibodies (a kind gift from G.B. Pier [39]). PNAG antibodies were detected using a secondary anti-goat antibody (dilution 1:5,000) conjugated with horseradish peroxidase. Immunoreactive spots were revealed using ECL Western blotting reagent (Amersham Pharmacia Biotech).

### Statistical analysis

When applicable, statistically significant differences among samples were determined using a *t*-test of analysis of variance (ANOVA) via a software run in MATLAB environment (Version 7.0, The MathWorks Inc.). Tukey’s honestly significant different test (HSD) was used for pairwise comparison to determine significance of the data. Statistically significant results were depicted by *p*-values <0.05.

## Results

### Lack of PNPase induces cell aggregation in *E*. *coli* C

**Figure 1:**
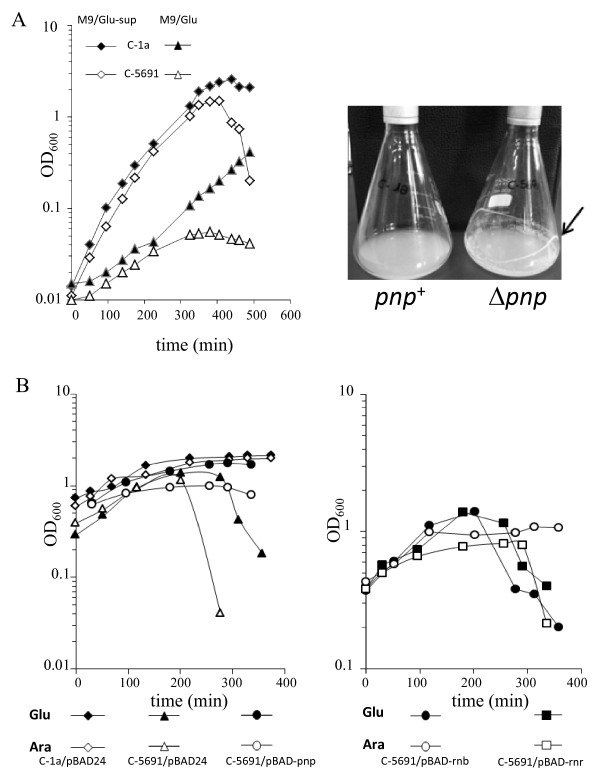
**Cell aggregation and adhesion by *E*. *coli *C PNPase-defective strain. A**. Growth curves of *E*. *coli* C-1a (*pnp*^+^; solid symbols) and *E*. *coli* C-5691 (Δ*pnp*-*751*; open symbols) in different media (M9Glu/sup, diamonds; M9Glu, triangles) (left panel). Cell clumping by the C-5691 (Δ*pnp*) strain led to deposition of ring-like aggregates on the flask walls (indicated by the arrow; right panel). The picture was taken in the late exponential phase (OD_600_ = 5–6). **B**. Cultures of strains carrying pBAD24 derivatives grown up to OD_600_ = 0.6-0.8 in M9Glu/sup at 37°C with aeration were harvested by centrifugation, resuspended in 0.04 vol M9 and diluted 25 fold in pre-warmed M9/sup with either 0.4% glucose (solid symbols) or 1% arabinose (empty symbols). Incubation at 37°C was resumed and growth monitored spectrophotometrically. Left panel: PNPase complementation. Right panel: suppression by RNase II.

The *E*. *coli* C *pnp* deletion mutant C-5691 (a derivative of *E*. *coli* C-1a [40,41]) showed an apparent growth arrest when grown at 37°C in M9 minimal medium with glucose as sole carbon source (M9Glu, Figure 1A, left panel). The growth defect was overcome by supplementing M9Glu with 0.25 g/l tryptone, 0.125 g/l yeast extract, 0.125 g/l NaCl (M9Glu/sup medium); however, in such conditions, C-5691 optical density drastically decreased at the onset of stationary phase. Such drop was due to cell flocculation, leading to formation of macroscopic cell clumps sedimenting onto the flask glass wall (Figure 1A, right panel). Cell flocculation also occurred when either arabinose or glycerol were added to M9/sup media instead of glucose (data not shown).

The aggregative phenotype of the C-5691 (Δ*pnp*) strain was complemented by basal expression from a multicopy plasmid of the *pnp* gene under *araBp* promoter, indicating that low PNPase expression is sufficient to restore planktonic growth. Conversely, arabinose addition did not completely restore a wild type phenotype (Figure 1B, left panel), suggesting that PNPase overexpression may also cause aggregation. Ectopic expression of RNase II suppressed the aggregative phenotype of the *pnp* mutant (Figure 1B, right panel), thus suggesting that such a phenotype is controlled by the RNA degrading activity of PNPase. In contrast, however, RNase R overexpression did not compensate for lack of PNPase, indicating that different ribonucleases are not fully interchangeable in this process.

### Inactivation of the *pnp* gene induces poly-*N*-acetylglucosamine (PNAG) production

**Figure 2:**
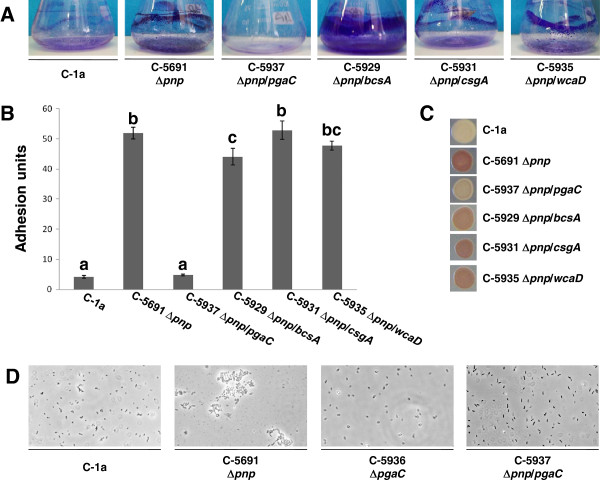
**Identification of the factor responsible for C-5691 (Δ*pnp*) aggregative phenotype. A**. Cell aggregation in C-1a (*pnp*^+^), C-5691 (Δ*pnp*) and C-5691 derivatives carrying mutations in genes encoding for adhesion determinants (Δ*pgaC*, C-5937; Δ*bcsA*, C-5929; Δ*csgA*, C-5931; Δ*wcaD*, C-5935). Cell aggregates were stained with crystal violet for better visualization. **B**. Surface adhesion of the same set of strains to polystyrene microtiter plates. The adhesion unit values, assessed as previously described [33], are the average of three independent experiments and standard deviation is shown. The overall *p*-value obtained by ANOVA was *p* = 5.11x10^-12^. Letters provide the representation for *posthoc* comparisons. According to *posthoc* analysis (Tukey’s HSD, *p* < 0.05), means sharing the same letter are not significantly different from each other. **C**. Phenotype on Congo red-supplemented agar plates. **D**. Phase contrast micrographs (1,000 x magnification) of *pnp*^+^ (C-1a), Δ*pnp* (C-5691), Δ*pgaC* (C-5936), and Δ*pnp* Δ*pgaC* (C-5937) strains grown overnight in M9Glu/sup medium at 37°C. The images were acquired with a digital CCD Leica DFC camera.

In addition to macroscopic cell aggregation (Figures 1 and 2A), deletion of *pnp* stimulated adhesion to polystyrene microtiter plates in a standard biofilm formation assay [33] (Figure 2B) and resulted in red phenotype on solid medium supplemented with Congo red, a dye binding to polymeric extracellular structures such as amyloid fibers and polysaccharides (Figure 2C). Cell aggregation was also observed by phase contrast microscopy (Figure 2D). Altogether, these observations strongly suggest that inactivation of *pnp* triggers the expression of one or more extracellular factors implicated in cell aggregation and adhesion to solid surfaces. In order to identify such factor(s), we searched for deletion mutants in genes encoding known adhesion factors and biofilm determinants that could suppress the aggregative phenotype of the C-5691 (Δ*pnp*) mutant strain. The following adhesion factors were targeted by appropriate mutations (Table 1): curli fibers (Δ*csgA*), which strongly promote attachment to abiotic surfaces and constitute the main determinant for Congo red binding [42,43]; cellulose (Δ*bcsA*) and PNAG (Δ*pgaA* and Δ*pgaC*), two extracellular polysaccharides able to promote surface adhesion and to affect Congo red binding to the bacterial cell [44,45]; and the capsular polysaccharide colanic acid (Δ*wcaD*), which promotes biofilm maturation acting synergistically with other adhesion factors such as curli fibers or conjugative pili [46,47].

The aggregative phenotype of the C-5691 (Δ*pnp*) mutant, as determined by cell aggregation, surface adhesion, and Congo red binding experiments, was totally abolished by deletion of *pgaC* (Figure 2), which encodes the polysaccharide polymerase needed for biosynthesis of PNAG from UDP-*N*-acetylglucosamine [48]. Deletion of *pgaA*, also part of the PNAG biosynthetic operon *pgaABCD*, produced identical effects as *pgaC* (data not shown). In contrast, no significant effects on either Congo red binding or cell aggregation and adhesion were detected in any Δ*pnp* derivative unable to produce curli or colanic acid (Figure 2). Finally, deletion of the *bcsA* gene, which encodes cellulose synthase, led to a significant increase in cell adhesion to the flask glass walls (Figure 2A); this result is consistent with previous observations suggesting that, although cellulose can promote bacterial adhesion, it can also act as a negative determinant for cell aggregation, particularly in curli-producing *E*. *coli* strains [49,50]. In the C-1a strain, carrying a wild type *pnp* allele, inactivation of genes involved in biosynthesis of curli, PNAG, cellulose and colanic acid did not result in any notable effects on cell aggregation (Additional file 2: Figure S1).

To establish whether induction of PNAG-dependent cell aggregation in the absence of PNPase is unique to *E*. *coli* C-1a or it is conserved in other *E*. *coli* strains, we performed adhesion assays comparing the standard laboratory strain MG1655 to its Δ*pnp* derivative KG206. Similar to what observed for the *E*. *coli* C strains, deletion of the *pnp* gene in the MG1655 background resulted in a significant increase in adhesion to solid surfaces, which was totally abolished by *pgaA* deletion (Additional file 3: Figure S2). However, cell aggregation was not observed in KG206 liquid cultures (data not shown), suggesting that the effect of *pnp* deletion is less pronounced in the MG1655 background.

**Figure 3:**
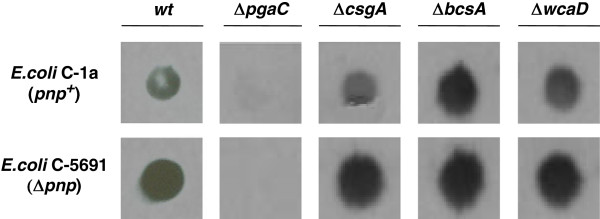
**Determination of PNAG production by immunological assay.** Crude extracts were prepared from overnight cultures grown in M9Glu/sup at 37°C. PNAG detection was carried out with polyclonal PNAG specific antibodies as detailed in Materials and Methods. PNAG determination was repeated four times (twice on each of two independent EPS extractions) with very similar results: data shown are from a typical experiment. Upper panel (*pnp*^+^): *E*. *coli* C-1a (wt), C-5936 (Δ*pgaC*), C-5930 (Δ*csgA*), C-5928 (Δ*bcsA*), C-5934 (Δ*wcaD*); lower panel (Δ*pnp*): *E*. *coli* C-5691 (wt), C-5937 (Δ*pgaC*), C-5931 (Δ*csgA*), C-5929 (Δ*bcsA*), C-5935 (Δ*wcaD*).

Our results clearly indicate that PNAG is required for the aggregative phenotype of *pnp* mutant strains, suggesting that PNPase may act as a negative regulator of PNAG production. We thus determined by western blotting PNAG relative amounts in both C-1a (WT) and C-5691 (Δ*pnp*) strains using anti-PNAG antibodies. As shown in Figure 3, the Δ*pnp* mutants (both with the single Δ*pnp* mutation and in association with either Δ*csgA* or Δ*wcaD*) exhibited higher PNAG levels relative to the *pnp*^+^ strains. As expected, no PNAG could be detected in *pgaC* mutants, whereas *bcsA* inactivation, which abolishes cellulose production, led to stimulation of PNAG biosynthesis. Despite increased PNAG production, the *pnp*^+^ Δ*bcsA* strain did not show any detectable cell aggregation (Additional file 2: Figure S1). Discrepancies between PNAG levels and aggregative phenotype in some mutants might be explained by presence of additional adhesion factors, or different timing in PNAG production.

### PNPase downregulates *pgaABCD* operon expression at post-transcriptional level

**Figure 4:**
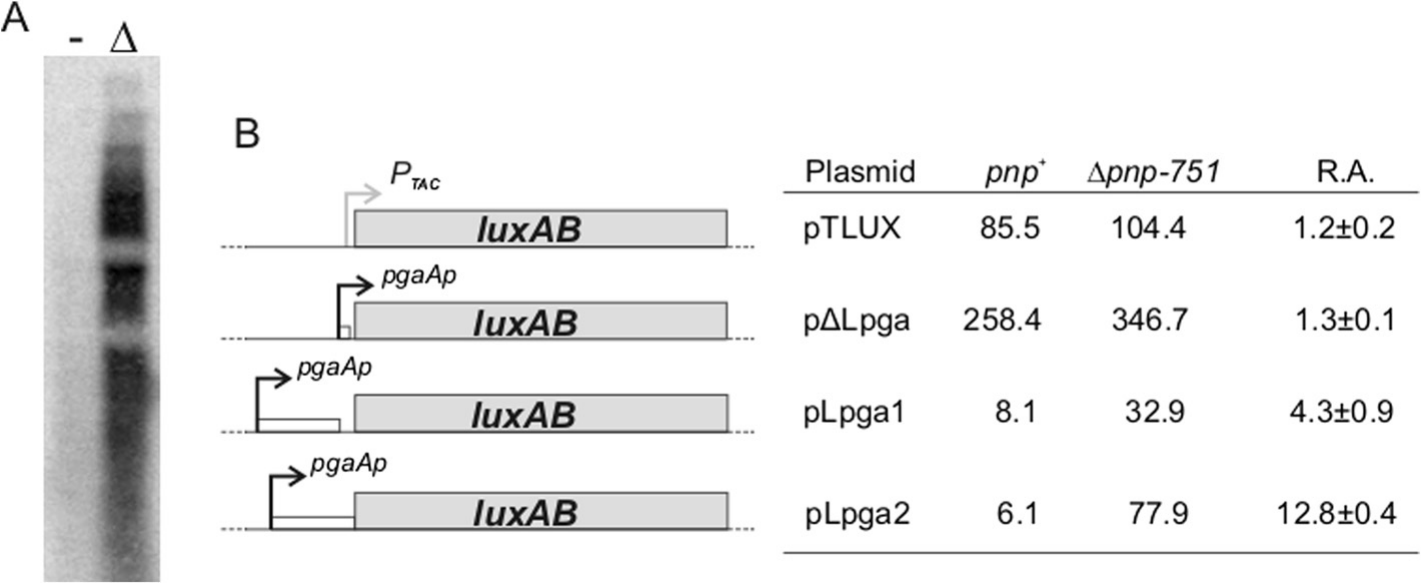
**Analysis of *pgaABCD *regulation by PNPase. A**. Northern blot analysis of *pgaABCD* operon transcription. 15 μg of total RNA extracted from *E*. *coli* C-1a ( *pnp*^+^) and *E*. *coli* C-5691 (Δ*pnp*-*751*) cultures grown up to OD_600_ = 0.8 in M9Glu/sup at 37°C were hybridized with the radiolabelled PGA riboprobe (specific for *pgaA*). **B**. Identification of in *cis* determinants of *pgaABCD* regulation by PNPase. Map of pJAMA8 luciferase fusion derivatives and luciferase activity expressed by each plasmid. Details about plasmid construction and coordinates of the cloned regions are reported in Methods and in Table 1. Construct elements are reported on an arbitrary scale. For relative luciferase activity (R.A.) in *E*. *coli* C-5691 (Δ*pnp*-*751*) *vs*. *E*. *coli* C-1a (*pnp*^+^) strains, average and standard deviation of at least two independent determinations are reported. Although the absolute values of luciferase activity could vary from experiment to experiment, the relative ratio of luciferase activity exhibited by strains carrying different fusions was reproducible. The results of a typical experiment of luciferase activity determination are reported on the right.

In *E*. *coli*, the functions responsible for PNAG biogenesis are clustered in the *pgaABCD* operon [48]. By northern blot analysis we found that the *pgaABCD* transcript was much more abundant in the Δ*pnp* strain than in *pnp*^+^ (Figure 4A), suggestive of negative control of *pgaABCD* transcript stability by PNPase. Increased transcription of the *pgaABCD* operon was also detected in the *E*. *coli* MG1655 Δ*pnp* derivative KG206 (data not shown), in agreement with biofilm formation experiments ( Additional file 3: Figure S2). We investigated the mechanism of *pgaABCD* regulation by PNPase and its possible connections with known regulatory networks controlling *pgaABCD* expression. *pgaABCD* expression is positively regulated at the transcription initiation level by NhaR, while *pgaABCD* mRNA stability and translation are negatively regulated by the CsrA protein [51,52]. The 234-nucleotide long *pgaABCD* 5’-UTR carries multiple binding sites for the translation repressor CsrA [51]. Two small RNAs, CsrB and CsrC, positively regulate *pgaABCD* by binding CsrA and antagonizing its activity [53]. Stability of the two small RNAs is controlled by CsrD, which triggers RNase E-dependent degradation by a still unknown mechanism [54]. Recently, a third sRNA, McaS, has been involved in this regulatory system as a positive regulator of *pgaABCD* expression [55].

Enhanced stability of *pgaABCD* mRNA may account for (or at least contribute to) the increase in *pgaABCD* expression. Indeed, RNA degradation kinetics experiments performed by quantitative RT-PCR showed a small, but reproducible 2.5-fold half-life increase of *pgaA* mRNA in the Δ*pnp* mutant (from 0.6 min in C-1a to 1.5 min in the *pnp* mutant; Additional file 4: Figure S3). A comparable effect was elicited by deletion of the *csrA* gene (estimated mRNA half-life, 1.5 min; Additional file 4: Figure S3), known to regulate *pgaABCD* mRNA stability in *E*. *coli* K12 [38,51].

Post-transcriptional regulation of the *pgaABCD* operon by the CsrA protein targets its 234 nucleotide-long 5’-UTR. Therefore, we tested whether this determinant was also involved in *pgaABCD* control by PNPase. To this aim, we constructed several plasmids (see Table 1) harboring both transcriptional and translational fusions between different elements of the *pgaABCD* regulatory region and the *luxAB* operon, which encodes the catalytic subunits of *Vibrio harveyi* luciferase, as a reporter [37]. Luciferase expression in both *pnp*^+^ and Δ*pnp* strains was tested using the transcriptional fusion plasmids pΔLpga and pLpga1, which harbor the *pgaABCD* promoter region (*pgaAp*) alone (−116 to +32 relative to the transcript start site) and a region encompassing *pgaAp* and the entire *pgaA* leader (without its ATG start codon), respectively. In these constructs, translation of the *luxAB* transcript depends on the vector translation initiation region (TIR). Conversely, pLpga2 carries a translational fusion of the whole 5’-UTR and the first 5 codons of *pgaA* with *luxA*. A plasmid expressing *luxAB* from *Ptac* promoter (pTLUX) and the vector TIR was also tested as a control of PNPase effects on luciferase mRNA expression. The results of a typical experiment and relative luciferase activity (Δ*pnp vs*. *pnp*^+^) are reported in Figure 4B. In agreement with the role of the 5’-UTR as a strong determinant for negative regulation of *pgaABCD* expression [51], luciferase activity was much higher in cells carrying the construct lacking the *pgaABCD* 5’-UTR (pΔLpga) regardless of the presence of PNPase. The small increment in luciferase expression from the pΔLpga plasmid detected in the Δ*pnp* was not due to increased *pgaAp* promoter activity as it was observed also with pTLUX control plasmid. Conversely, luciferase expression by pLpga1 and pLpga2 was strongly affected by PNPase, as it increased 4.3- and 12.8-fold, respectively, in the PNPase defective strain (Figure 4B). The difference in relative luciferase activity between the pLpga1 and pLpga2 constructs might be explained by higher translation efficiency for the pLpga2 construct in the Δ*pnp* strain. Altogether, the results of luciferase assays (Figure 4B) and mRNA decay experiments (Additional file 4: Figure S3) suggest that PNPase regulates *pgaABCD* mRNA decay by interacting with *cis*-acting determinants located in the 5’-UTR. PNPase has been recently shown to play a pivotal role in sRNA stability control [27,56] and has been involved in degradation of CsrB and CsrC in *Salmonella*[57]. We hypothesized that PNPase may act as a negative regulator of *pgaABCD* operon by promoting the degradation of the positive regulators CsrB and/or CsrC [53]. To test this idea, we combined the Δ*pnp751* mutation with other deletions of genes either encoding sRNAs known to affect *pgaABCD* expression (namely, *csrB*, *csrC* and *mcaS*), or *csrD*, whose gene product favors CsrB and CsrC degradation [54]. We also readily obtained the Δ*csrA*::*kan* mutation in C-1a (*pnp*^+^), indicating that, unlike in K-12 strains [58], *csrA* is not essential in *E*. *coli* C. Conversely, in spite of several attempts performed both by λ Red mediated recombination [32] and by P1 reciprocal transductions, we could not obtain a Δ*pnp* Δ*csrA* double mutant, suggesting that the combination of the two mutations might be lethal.

**Figure 5:**
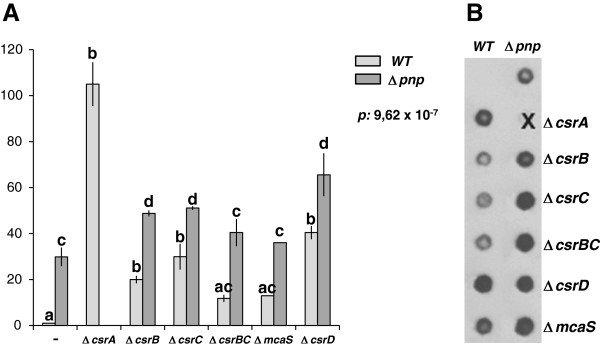
***pgaABCD *expression in mutants defective for CsrA-dependent regulation elements and/or PNPase.** See Table 1 for the complete list of strains used in these experiments. A Δ*pnp* Δ*csrA* double mutant could not be obtained. **A**. *pgaABCD* mRNA expression. RNA was extracted from cultures grown in M9Glu/sup to OD_600_ = 0.8 and analyzed by quantitative RT-PCR as described in Methods. White bars, *pnp*^+^ strains; dark grey, Δ*pnp* strains. The “Relative expression” values indicated in the graph are the average of three independent experiments, each performed in duplicate, and standard deviations are shown. The overall *p*-value obtained by ANOVA is indicated in the graph. Letters provide the representation for *posthoc* comparisons. According to *posthoc* analysis (Tukey’s HSD, *p* < 0.05), means sharing the same letter are not significantly different from each other. **B**. PNAG production. Crude extracts from overnight cultures were filtered onto a nitrocellulose membrane, and PNAG detection was carried out using polyclonal PNAG specific antibodies as detailed in Materials and Methods. PNAG determination was repeated at least four times on three independent EPS extractions with comparable results; data shown are from a typical experiment.

Each mutant was assayed for the expression of *pgaA* by quantitative RT-PCR and for PNAG production by western blotting. The results of these analyses showed that, both in the C-1a (*pnp*^+^) and in the C-5691 (Δ*pnp*) backgrounds, each tested mutation increased both *pgaA* mRNA expression (Figure 5A) and PNAG production (Figure 5B). This result was unexpected for mutants lacking CsrB, CsrC or McaS that, according to the current model of *pgaABCD* regulation, should act as positive regulators of such operon [51]. Thus, while our results support the role of CsrA as a major regulator of *pgaABCD* expression, they also suggest that the current model for *pgaABCD* post-transcriptional regulation, which is based on data obtained in *E*. *coli* K-12, may not readily apply to *E*. *coli* C. The additive effect observed upon combining Δ*pnp751* with deletions targeting different sRNAs suggest that PNPase and the sRNAs may act independently on *pgaABCD* regulation.

## Discussion

In this report, we have shown that PNPase negatively regulates the production of the adhesion factor PNAG, thus maintaining the bacterial cells in a planktonic state (Figures 1,3) when grown at 37°C in supplemented minimal medium. Our results are in line with previous works by other groups connecting PNPase to regulation of outer membrane proteins in *E*. *coli*[59] and curli production in Salmonella [60]. Thus, PNPase seems to play a pivotal role in regulating the composition of cell envelope and the production of adhesion surface determinants. PNPase-dependent regulation of PNAG production requires its ribonuclease activity, as suggested by the observation that overexpression of RNase II can compensate for lack of PNPase (Figure 1B). Cell aggregation in the absence of PNPase is suppressed by RNase II, but not by RNase R. This reminds what previously showed for cold sensitivity in *pnp* mutants, which is also solely suppressed by RNase II [61] and reinforces the notion that, albeit partially redundant, RNA degradation pathways possess a certain degree of specificity and are not fully interchangeable [62].

The precise mechanistic role played by PNPase in regulation of *pgaABCD* expression, as well as the physiological signals to which it responds, remain elusive. PNPase activity is modulated (at least *in vitro*) by cyclic-di-GMP [63], a signal molecule implicated in biofilm formation [18]. However, deletion of the *dos* gene, encoding a c-di-GMP phosphodiesterase which co-purifies with the RNA degradosome [63], did not affect *pgaABCD* expression (data not shown). Key molecules in energy metabolism and carbon flux, such as ATP and citrate also influence PNPase activity [64,65]. Thus, it can be speculated that environmental or physiological signals might regulate *pgaABCD* expression by controlling the level of specific metabolites that could directly modulate PNPase activity.

Our data clearly indicate that PNPase controls PNAG production by negatively regulating the *pgaABCD* operon at post-transcriptional level and that it targets the 5’-UTR of the *pgaABCD* transcript, thus similar to the translational repressor CsrA (Figures 4,5 and Additional file 4: Figure S3). This would suggest that the two proteins might belong to the same regulatory network. However, probing this hypothesis is complicated by the observation that in *E*. *coli* C, the mechanisms of CsrA-dependent gene expression regulation and its modulation by small RNAs might be more complex than in *E*. *coli* K-12, where the current model for CsrA regulation has been developed. This notion is somehow suggested by the fact that, while deletion of the *csrA* gene is lethal for *E*. *coli* K-12 when grown on glucose-based media [55], this is not the case for *E*. *coli* C. Moreover, to our surprise, the lack of putative positive regulators such as CsrB, CsrC and McsA resulted in an increase of *pgaABCD* expression levels both in the Δ*pnp* and in its parental strain C-1a, which would suggest a negative role of these sRNAs in *pgaABCD* control (Figure 5). Genes encoding cell surface-associated structures seem to constitute a “hotspot” for post-transcriptional regulation involving small non coding RNAs. For instance, multiple control of gene expression by sRNAs has already been demonstrated for *csgD*, which encodes the master regulator for the biosynthesis of thin aggregative fimbriae (curli), one of the major adhesion factors in *E*. *coli*[28,55,66,67]. It is thus possible that, in *E*. *coli* C, increased *pgaABCD* expression in mutant strains carrying deletions of sRNA-encoding genes might be due to feedback induction of yet unidentified factors which might play a role in CsrA-dependent regulation. This possibility is supported by the observation that CsrB, CsrC and McaS mutually control their transcript level both in *E*. *coli* K and C [53] (T. Carzaniga and F. Briani, unpublished data). *pgaABCD* operon regulation appears to be an intriguing model system for the study of post-transcriptional modulation of gene expression in bacteria.

## Conclusions

In this work, we have unravelled a novel role for PNPase as a negative regulator of *pgaABCD* expression and PNAG biosynthesis. Thus, PNPase activity contributes to keeping *E*. *coli* cells in the planktonic state. Our findings underline the importance of post-transcriptional regulation for genes encoding cell surface-associated structures and factors involved in biofilm formation and suggest the existence of strain-specific variability in these regulatory mechanisms. Indeed, small RNA-dependent post-transcriptional regulation of *pgaABCD* expression in *E*. *coli* C is more complex than the model proposed for *E*. *coli* K-12, possibly connected to a central role played by PNAG as a determinant for biofilm formation in the former strain.

## Authors’ contributions

FB, GD and PL conceived the project and designed the experiments. FB and PL wrote the manuscript. TC and DA designed and performed the experiments. All authors read and approved the final manuscript.

## Supplementary Material

Additional file 1: Table S1 Primers used in this work. Click here for file

Additional file 2: Figure S1 Effects of inactivation of genes encoding adhesion factors and biofilm determinants in the C-1a strain. C-1a (*pnp*^+^) and its derivatives carrying mutations in genes encoding for adhesion determinants (Δ*pgaC*, impaired in PNAG production; Δ*bcsA*, impaired in cellulose production; Δ*csgA*, impaired in curli production; Δ*wcaD*, impaired in colanic acid production) were grown over night in M9Glu/sup at 37°C in glass flasks. Cell aggregates were stained with crystal violet. Click here for file

Additional file 3: Figure S2 Surface adhesion of *pnp* deletion mutant derivative of *E*. *coli* MG1655 and identification of the adhesion factor involved. Surface adhesion to polystyrene microtiter plates by MG1655 (*pnp*+), KG206 (Δ*pnp*), and KG206 derivatives carrying mutations in genes coding for adhesion determinants (Δ*pgaA*, AM56; Δ*bcsA*, AM72; Δ*csgA*, AM70; Δ*wcaD*, AM105) was assessed at 37°C in M9Glu/sup. Adhesion unit values, assessed as previously described [33], are the average of three independent experiments and standard deviation is shown. The overall *p*-value obtained by ANOVA is indicated in the graph. Letters provide the representation for *posthoc* comparisons. According to *posthoc* analysis (Tukey’s HSD, *p* < 0.05), means sharing the same letter are not significantly different from each other. Click here for file

Additional file 4: Figure S3 *pgaA* mRNA decay analysis. Bacterial cultures of C-1a (*pnp*^+^), C-5691 (Δ*pnp*) and C-5938 (Δ*csrA*) were grown up to OD_600_ = 0.8 in M9Glu/sup, rifampicin (final concentration of 0.4 mg/ml) was added, and samples for RNA extraction were taken at different time points immediately before (t = 0) and after antibiotic addition. *pgaA* mRNA degradation kinetics was estimated by quantitative RT-PCR with oligonucleotides PL99 and PL100, as detailed in Methods. Click here for file
