# Supplementary material for: The RNA processing enzyme polynucleotide phosphorylase negatively controls biofilm formation by repressing poly-N-acetylglucosamine (PNAG) production in Escherichia coli C
Source: BMC Microbiol. 2012 Nov 21;12:270. doi: 10.1186/1471-2180-12-270 (PMC3571907; doi:10.1186/1471-2180-12-270)
Supplement: Additional file 1: Table S1 — Primers used in this work. [file 1471-2180-12-270-S1.docx]

**Supplementary Table 1**

| **Oligonucleotide** | | **5′→3′ sequence** | **Coordinates^a^** |
| --- | --- | --- | --- |
| FG2474/31 | *CATGCCATGG*GCTGGTGGAGTGACGAAAATC | | *NcoI site*/ 4404626-4404646- |
| FG2475/29 | *ACAAAGCTT*TCCGGCATGAACAAAGCGCA | | *HindIII* *site*/ 4407162-4407181 |
| FG2624/70^b^ | CGGGATACAGAGAGACCCGACTCTTTTAATCTTTCAAGGAGCAAAGAATG**attccggggatccgtcgacc** | | 2817166-2817215  /**1313-1334** |
| FG2625/70^b^ | CTCACCGATAAAGATGAGACGCGGAAAGATTAGTAACTGGACTGCTGGGA**tgtaggctggagctgcttcg** | | 2816954-2817003  /**32-51** |
| FG2524/70^b^ | AGCGCCTTGTAAGACTTCGCGAAAAAGACGATTCTATCTTCGTCGACAGG**tgtaggctggagctgcttcg** | | 2922538-2922587  /**32-51** |
| FG2525/70^b^ | AGGGAGCACTGTATTCACAGCGCTCCCGGTTCGTTTCGCAGCATTCCAGC**attccggggatccgtcgacc** | | 2922187-2922236  /**1313-1334** |
| FG2585/70^c^ | AAAATGTGCAAATACTGATGGCGGTTGATTGTTTGTTTAAAGCAAAGGCG**tgtgtaggctggagctgctt** | | 4048996-4049045  /**30-49** |
| FG2586/70^c^ | TGCGGCGGAATCTAACAGAAAGCAAGCAAAGAAAAAAGGCGACAGATTAA**CATATGAATATCCTCCTTAG** | | 4049283-4049331  /**1488-1507** |
| PL674/70^c^ | TGATTTGCTAGTATGCCCGCTTCCTCACTATCGGAGTTAACACAAGGATG**TGTGTAGGCTGGAGCTGCTT** | | 3401352-3401401  /**30-49** |
| PL675/70^c^ | TTCTACGTGAAAACGGATTAAACGGCAGGTTAAACCGAGTATCTTTGTGA**CATATGAATA TCCTCCTTAG** | | 3399385-3399434  /**1488-1507** |
| PL372/60^e^ | AGCCGCGCCGCCTACAGTGGACAACAGATGCTGGAGGAGT**CTAGACATCATTAATTCCTA** | | 2128943-2128902  /**5318-5337** |
| PL373/60^e^ | ATGCTAACGGTAACTTTTTCTTAATCGCCTCTTTATTCAG**GAAGCTAAATCTTCTTTATCG** | | 2118203-2128163  /**6686-6706** |
| FG2755/70^c^ | AGTTAAAACTGCATAAAAAAATAGAGTCTGTCGACATCCGCCAGACTCTA**ATTCCGGGGATCCGTCGACC** | | 1403668-1403717  /**1313-1334** |
| FG2756/70^c^ | ACTGAAGAAAATTGGCAACTAAAGGTTAAAACCGTTATAACACAGTCACC**TGTAGGCTGGAGCTGCTTCG** | | 1403773-1403822  /**32-51** |
| FG2491/39^d^ | **CTAATACGACTCACTATAGGG**CGTTGCGCCAGCATCAAT | | 1089170 -1089187 |
| FG2492/22 | GATATTGTTCCGGCATTTGAGG | | 1089331-1089352 |
| PL99/21 | CCGCTACCGTCATCAGCAATT | | 1091779-1091790 |
| PL100/20 | AGCGCCTTTTGCCACAGTGT | | 1091867-1091887 |
| PL101/21 | TGTCGTCAGCTCGTGTCGTGA | | 4034614-4034625 |
| PL102/19 | ATCCCCACCTTCCTCCGGT | | 4034725-4034744 |

^a^Co-ordinates are referred to NCBI Accession Number U00096.2.

^b^Boldface letters and coordinates are from the pKD13 plasmid (NCBI Accession Number AY048744.1).

^c^Boldface letters and coordinates are from the pKD3 plasmid (NCBI Accession Number AY048742.1).

^d^Boldface letters indicate T7 promoter sequence.

^e^ Boldface letters and coordinates are from the transposon Tn10 (NCBI Accession Number AF162223).
