# Supplementary figures and images for: The RNA processing enzyme polynucleotide phosphorylase negatively controls biofilm formation by repressing poly-N-acetylglucosamine (PNAG) production in Escherichia coli C
Source: BMC Microbiol. 2012 Nov 21;12:270. doi: 10.1186/1471-2180-12-270 (PMC3571907; doi:10.1186/1471-2180-12-270)

## Slide 1
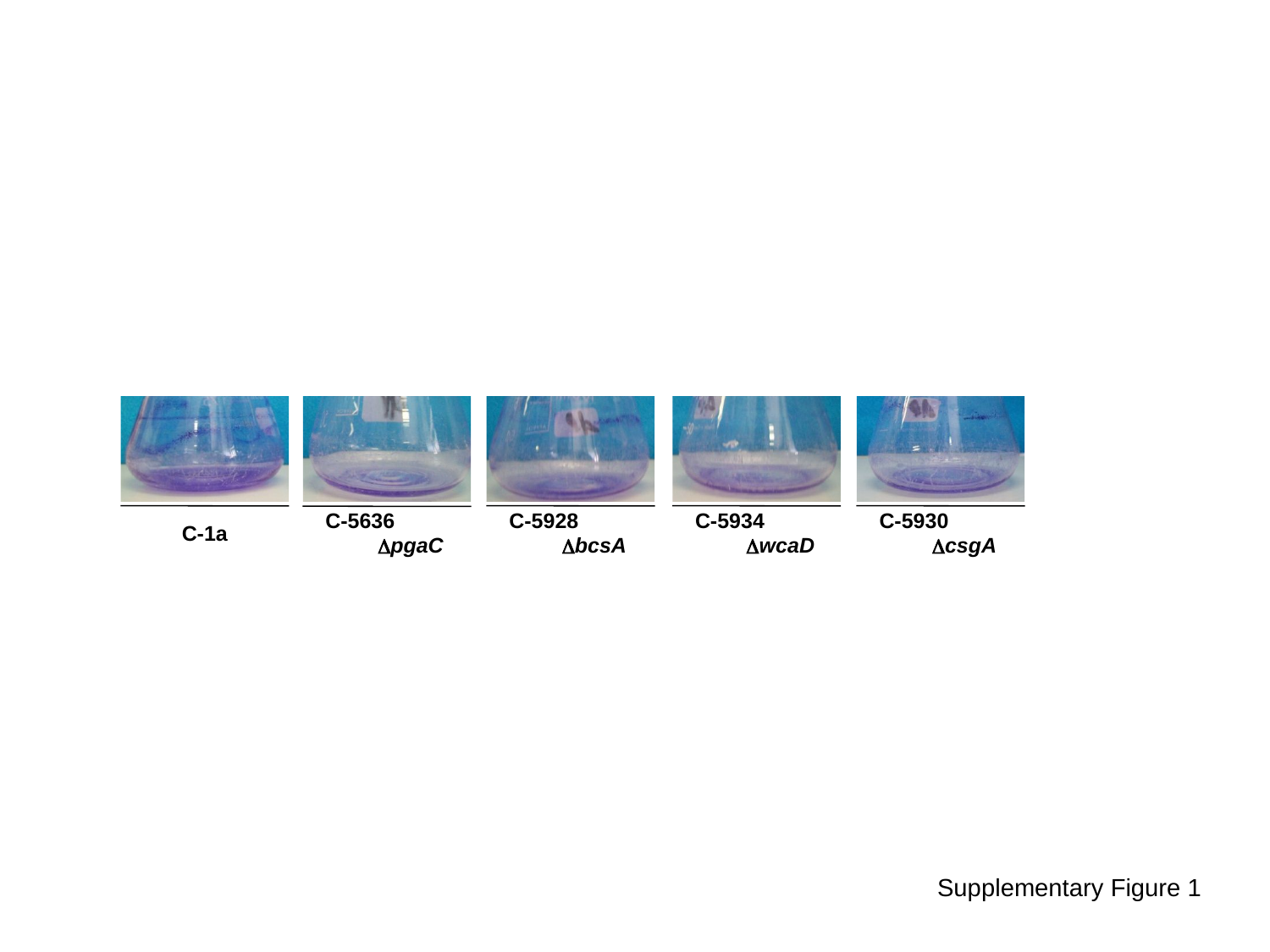

C-5636 DpgaC
C-5928 DbcsA
C-5934 DwcaD
C-5930 DcsgA
C-1a
Supplementary Figure 1

Supplement: Additional file 2: Figure S1 — Effects of inactivation of genes encoding adhesion factors and biofilm determinants in the C-1a strain. C-1a (pnp+) and its derivatives carrying mutations in genes encoding for adhesion determinants (ΔpgaC, impaired in PNAG production; ΔbcsA, impaired in cellulose production; ΔcsgA, impaired in curli production; ΔwcaD, impaired in colanic acid production) were grown over night in M9Glu/sup at 37°C in glass flasks. Cell aggregates were stained with crystal violet. [file 1471-2180-12-270-S2.pptx]

## Slide 1
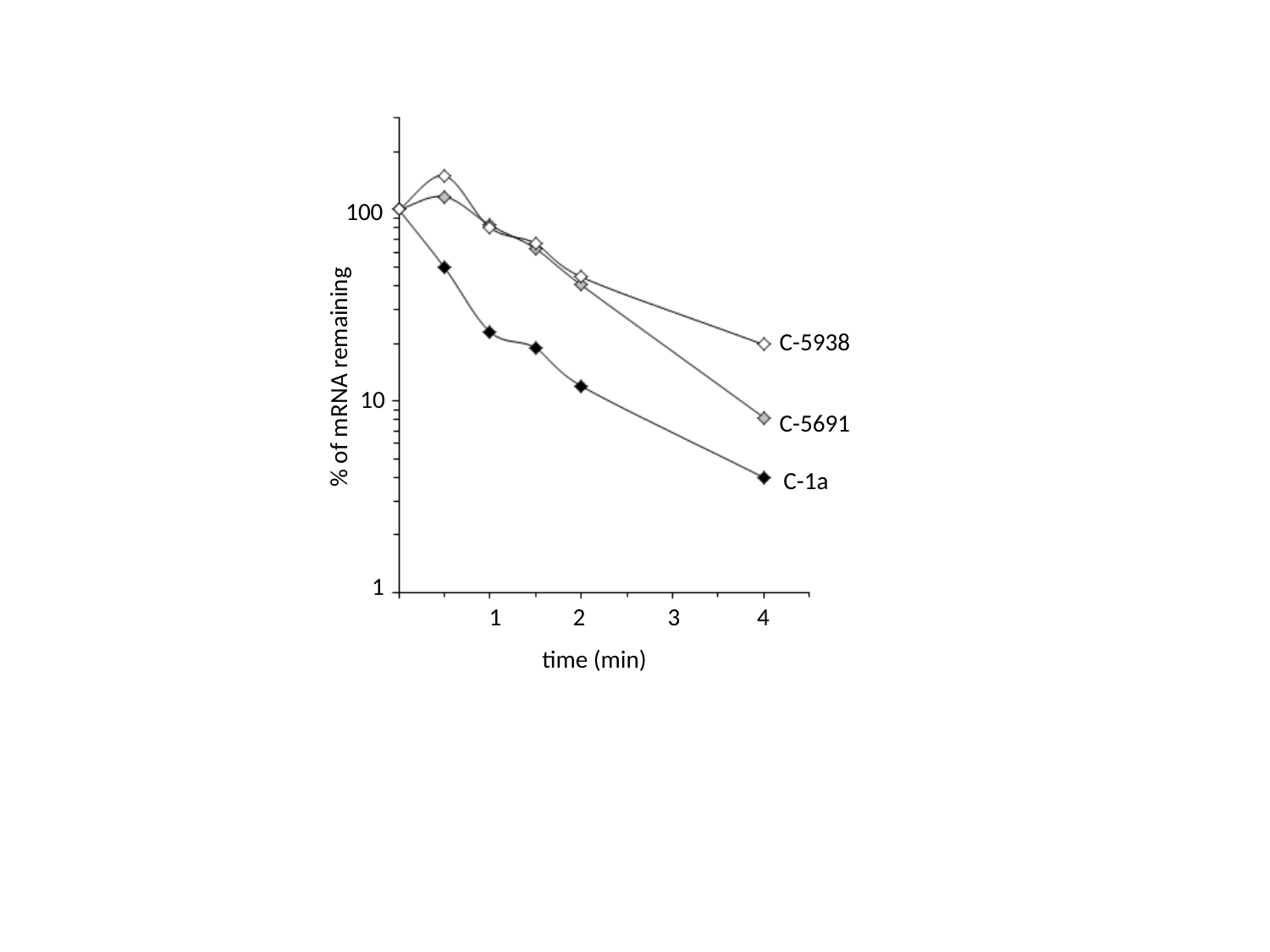

100
C-5938
% of mRNA remaining
10
C-5691
C-1a
1
 1 2 3 4
time (min)

Supplement: Additional file 4: Figure S3 — pgaA mRNA decay analysis. Bacterial cultures of C-1a (pnp+), C-5691 (Δpnp) and C-5938 (ΔcsrA) were grown up to OD600 = 0.8 in M9Glu/sup, rifampicin (final concentration of 0.4 mg/ml) was added, and samples for RNA extraction were taken at different time points immediately before (t = 0) and after antibiotic addition. pgaA mRNA degradation kinetics was estimated by quantitative RT-PCR with oligonucleotides PL99 and PL100, as detailed in Methods. [file 1471-2180-12-270-S4.pptx]
