# Supplementary material for: The RNA processing enzyme polynucleotide phosphorylase negatively controls biofilm formation by repressing poly-N-acetylglucosamine (PNAG) production in Escherichia coli C
Source: BMC Microbiol. 2012 Nov 21;12:270. doi: 10.1186/1471-2180-12-270 (PMC3571907; doi:10.1186/1471-2180-12-270)
Supplement: Additional file 3: Figure S2 — Surface adhesion of pnp deletion mutant derivative of E. coli MG1655 and identification of the adhesion factor involved. Surface adhesion to polystyrene microtiter plates by MG1655 (pnp+), KG206 (Δpnp), and KG206 derivatives carrying mutations in genes coding for adhesion determinants (ΔpgaA, AM56; ΔbcsA, AM72; ΔcsgA, AM70; ΔwcaD, AM105) was assessed at 37°C in M9Glu/sup. Adhesion unit values, assessed as previously described [33], are the average of three independent experiments and standard deviation is shown. The overall p-value obtained by ANOVA is indicated in the graph. Letters provide the representation for posthoc comparisons. According to posthoc analysis (Tukey’s HSD, p < 0.05), means sharing the same letter are not significantly different from each other. [file 1471-2180-12-270-S3.pptx]

## Slide 1
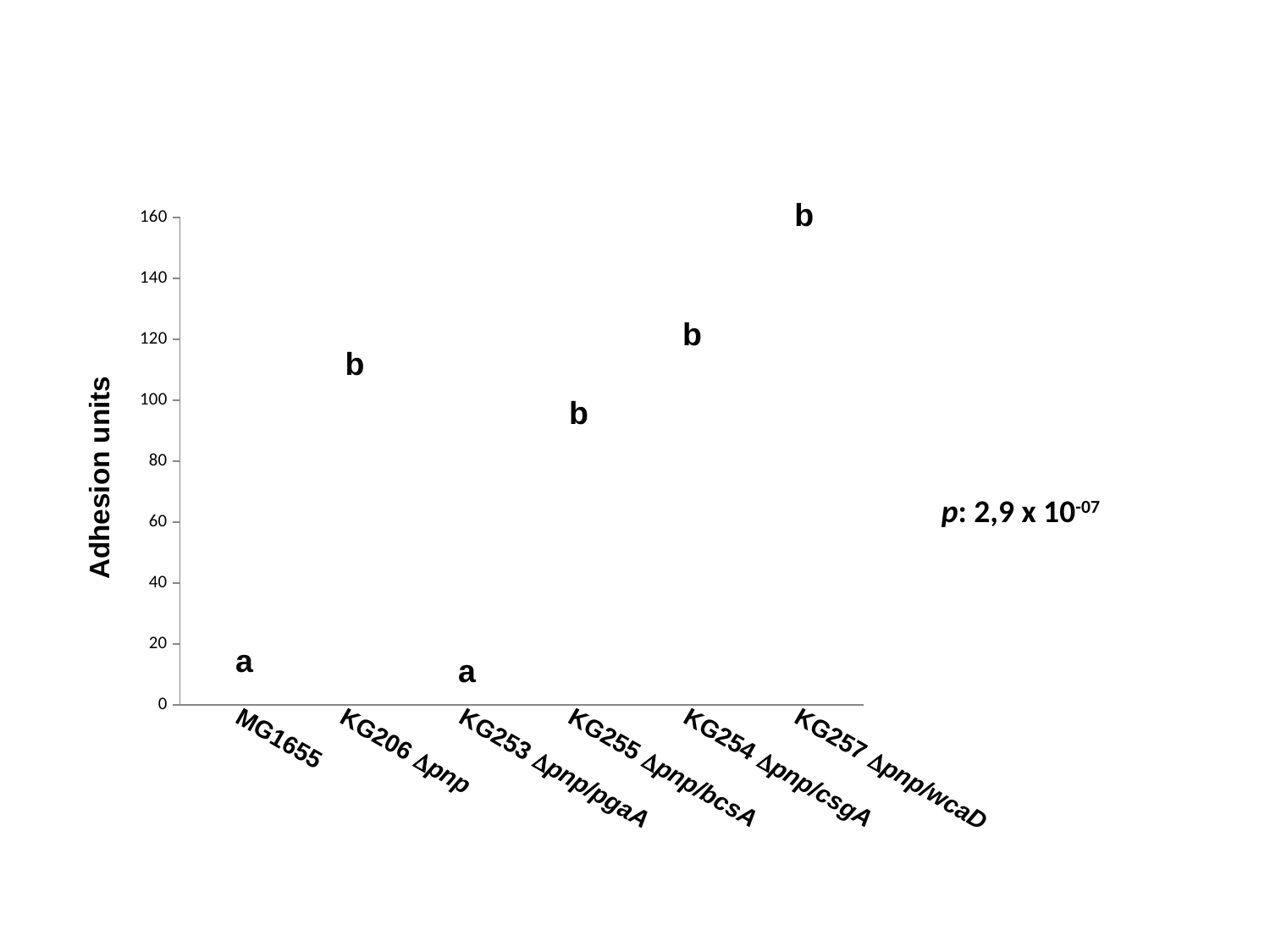

b
### Chart
| Category | |
|---|---|
| MG1655 | 4.335 |
| KG206 | 96.46000000000002 |
| KG206 ycdS::cam | 1.28 |
| KG206 bcsA::cam | 84.80000000000001 |
| KG206 csgA::cam | 98.915 |
| KG206 wcaD::tet | 110.0 |b
b
b
Adhesion units
p: 2,9 x 10^-07^
a
a
MG1655
KG206 Dpnp
KG253 Dpnp/pgaA
KG255 Dpnp/bcsA
KG254 Dpnp/csgA
KG257 Dpnp/wcaD
